# Human Mycotoxin Biomonitoring: Conclusive Remarks on Direct or Indirect Assessment of Urinary Deoxynivalenol

**DOI:** 10.3390/toxins12020139

**Published:** 2020-02-24

**Authors:** Arnau Vidal, Nabila Bouzaghnane, Sarah De Saeger, Marthe De Boevre

**Affiliations:** Centre of Excellence in Mycotoxicology and Public Health, Department of Bioanalysis, Ghent University, Ottergemsesteenweg 460, 9000 Ghent, Belgiumsarah.desaeger@ugent.be (S.D.S.); marthe.deboevre@ugent.be (M.D.B.)

**Keywords:** risk assessment, deoxynivalenol, glucuronides, indirect, glucuronidase, LC–MS/MS

## Abstract

Deoxynivalenol is one of the most ubiquitous mycotoxins in the Western diet through its presence in cereals and cereal products. A vast amount of studies indicate the worrying level of exposure to this toxin, while even high percentages of the population exceed the tolerable daily intake. To evaluate and assess dietary exposure, analysis of urinary levels of deoxynivalenol and its glucuronides has been proposed as a reliable methodology. An indirect preliminary method was used based on the cleavage of deoxynivalenol glucuronides through the use of enzymes (β-glucuronidase) and subsequent determination of "total deoxynivalenol" (sum of free and released mycotoxins by hydrolysis). Next, a direct procedure for quantification of deoxynivalenol-3-glucuronide and deoxynivalenol-15-glucuronide was developed. As deoxynivalenol glucuronides reference standards are not commercially available, the indirect method is widely applied. However, to not underestimate the total deoxynivalenol exposure in urine, the direct and indirect methodologies need to be compared. Urinary samples (n = 96) with a confirmed presence of deoxynivalenol and/or deoxynivalenol glucuronides were analysed using both approaches. The indirect method clarified that not all deoxynivalenol glucuronides were transformed to free deoxynivalenol during enzymatic treatment, causing an underestimation of total deoxynivalenol. This short communication concludes on the application of direct or indirect assessment of urinary deoxynivalenol.

## 1. Introduction

Deoxynivalenol (DON) is a food contaminant mycotoxin that produces a potent inhibition of protein synthesis. DON presence causes the pro-inflammatory response, produces ribotoxic stress, cytotoxicity and apoptosis and ends in the impairment of various physiological functions, such as the gastrointestinal mucosa, growth, immune regulation and reproduction [1,2,3]. Furthermore, this mycotoxin has been associated with acute animal and human gastro-enteritis outbreaks [4]. DON, as all trichothecenes, is commonly found in cereals and cereal-based products [5,6]; therefore, these foodstuffs are the principal contributors to chronic dietary exposure to DON and its metabolites [7]. Additionally, recent studies revealed that the mean attributed chronic dietary exposure exceeds the group tolerable daily intake (group-TDI) of 1 µg/kg body weight/day in children (including infants and toddlers), and a high exposure was observed in both adolescents and adults [8]. Additionally, our group recently revealed an association between chronic DON-exposure and colorectal cancer risk [9], which is in line with the findings of Payros et al. [10,11]. These facts point out a potential health concern, although IARC Monographs attributed that DON has no evidence for carcinogenicity to humans [7,12,13,14].

To fully unravel exposure to DON, the analysis of DON in urine has been proposed due to its fast excretion [15]. A few published results revealed that deoxynivalenol glucuronides, which are the main phase II metabolites of DON, are validated biomarkers in urine, namely deoxynivalenol-3-glucuronide (DON-3-glucuronide) and deoxynivalenol-15-glucuronide (DON-15-glucuronide) [16]. Approximately 90% of urinary DON is conjugated with glucuronic acid [15]. To determine the urinary glucuronides, an indirect method was developed based on the enzymatic hydrolysis of DON glucuronides and subsequent determination of "total DON" (sum of free and modified mycotoxins by hydrolysis) [17]. Next, a direct method for quantification of glucuronides (DON-3-glucuronide and DON-15-glucuronide) was described using in-house synthesized mycotoxin-standards [15]. These analytical advances allowed the scientific community to observe a high correlation between the sum of urinary DON and its glucuronides [18]. However, the lack of commercial standards for DON-glucuronides further cause limitations to accurately quantify DON exposure.

Although both methods, direct and indirect, have been used in several publications, the methods have never been compared to our knowledge. For this reason, the main aim of this study was to analyse DON, DON-3-glucuronide, DON-15-glucuronide, deoxynivalenol-3-glucoside (DON-3-glucoside), deepoxy-deoxynivalenol (DOM-1), 3-acetyldeoxynivalenol (3-ADON) and 15-acetyldeoxynivalenol (15-ADON) in urinary samples of DON-exposed human volunteers using both methodologies.

## 2. Results

The detected increase of free DON (from 43% to 71% > LOQ) and concentration (from 4.18 ± 2.63 nmol of free DON to 23.89 ± 8.61 nmol of free DON) that took place in the urine after enzymatic treatment was expected (Table 1). During enzymatic treatment, β-glucuronidase converted DON glucuronide forms into free DON.

Deoxynivalenol glucuronides forms (DON-15-glucuronide and DON-3-glucuronide) had lower presence when the sample was submitted to enzymatic treatment, especially DON-3-glucuronide (Table 1). During the analyses, 72.4% and 74.4% of DON-3-glucuronide and DON-15-glucuronide, respectively, were transformed to free DON. However, enzymatic treatment could not convert all glucuronide forms to free DON. DON-3-glucuronide was completely removed in 67% of the samples, but DON-15-glucuronide was only removed in three samples (the three samples had initially <1 nmol of DON-15-glucuronide). The reduction was larger for DON-3-glucuronide, probably because the initial concentration of DON-15-glucuronide was four-fold higher than DON-3-glucuronide concentration. The larger concentration of DON-15-glucuronide was expected, as DON-15-glucuronide is the predominant DON metabolite in human urine [15]. There was not any statistical difference (*p* < 0.05) in total DON presence between the indirect and direct method; however, the average concentration was lower in the indirect (27.69 ± 26.64 nmol) than in the direct method (37.95 ± 37.21 nmol). After enzymatic treatment, some DON glucuronides (DON-15-glucuronide or DON-3-glucuronide) were not converted to DON, so there was an underestimation of the total DON and as a result an underestimation of the DON-exposure in the urine when samples were only analysed by the indirect method.

DOM-1 traces were found in two samples (<LOQ), and no changes were detected between the indirect and direct method. The low presence of DOM-1 was expected because it is a residual metabolic transformation product of DON in human urine [8], and it is a predominant compound in faeces because it is produced by gastro-intestinal bacteria [19]. Due to the low concentration of DOM-1 in human urine, DOM-1 is not recommended as a trustworthy urinary biomarker for DON exposure. Six different DOM-1-glucuronides have been identified in vitro and in vivo studies using humans and animals (deepoxy-DON-15-glucuronide, deepoxy-DON-3-glucuronide, iso-deepoxy-DON, iso-deepoxy-DON-15-glucuronide, iso-deepoxy-DON-3-glucuronide and iso-deepoxy-DON-8-glucuronide) [16,20,21,22], but only deepoxy-DON-15-glucuronide, deepoxy-DON-3-glucuronide and iso-deepoxy-DON-3-glucuronide were detected in animal urine [20]. Deepoxy-DON-3-glucuronide was considered as a predominant compound in cow urine, so it could be possible that the ruminant’s gastro intestinal tract leads to an increase of deepoxy-DON forms. In pig urine, only minor levels of deepoxy-DON-15-glucuronide were verified [20]. So, it is expected that the deepoxy-DON glucuronides are not predominant in human urine. The enzymatic treatment could cause an increase of DOM-1 due to the presence of the DOM-1 glucuronide forms, but the possible low concentration of DOM-1 (<LOQ) made it difficult to identify an increase of DOM-1.

Several publications describe an indirect approach to estimate total DON in urine (Table 2). Unfortunately, more of these studies analysed DON glucuronides as a comparison. Moreover, every study used different enzymatic treatment conditions (different enzyme, pH and amount of enzyme). Only one study compared the direct and indirect methodologies with similar results for both methods: 20.4 ± 2.4 ng total DON/mL (direct) and 19.5 ± 1.2 ng total DON/mL (indirect) [23]. However, DON glucuronides were not analysed after the indirect method. Differences regarding the enzyme type were detected [24]. β-glucuronidase from *Helix pomatia* (Type I) and *Escherichia coli* (Type IX) showed better performances than *Patella vulgate* (Type L-II). The enzyme concentration did not affect the conversion rate; only free DON was higher (9%) when 10,000 units of β-glucuronidase from *Escherichia coli* were applied instead of 5000 units.

## 3. Conclusions

During the last decade, more focus has been set towards the assessment of human mycotoxin exposure through the analysis of biomarkers of exposure. Due to its common presence in cereal products, DON has been widely analysed in urine, and several publications estimated DON-exposure through urinary biomarkers. Most of the studies indicated a chronic exposure to DON, and several of them demonstrated that the population exceeds the TDI (1 µg/kg bw/day) [12,13,17]. To correctly assess urinary DON, it is imperative to quantify free DON, DON-3-glucuronide and DON-15-glucuronoide, which are the main DON biomarkers [15]. For this reason, it is crucial to develop a robust quantification method for these compounds in the urine to correctly assess DON exposure. The no-transformation of all DON glucuronides to free DON observed in the indirect method could cause an underestimation of the DON exposure. For this reason, this study suggests the use of the direct method to quantify total DON in urine because it will permit to achieve the most accurate estimation of total DON presence in urine. However, the lack of commercial standards for DON glucuronides still provokes difficulties for quantification.

## 4. Materials and Methods

### 4.1. Reagents and Chemicals

The individual mycotoxin solid calibration standards (1 mg) of DON, 3-ADON, 15-ADON, DOM-1, DON-3-glucoside, isotope-labelled (^13^C_15_) deoxynivalenol (^13^C_15_-DON) (internal standard) and β-glucuronidase from *Helix pomatia* Type H-2 (≥85,000 units/mL) were obtained from Sigma Aldrich (Bornem, Belgium). The mycotoxin standards were dissolved in methanol (1 mg/mL) and were stable for a minimum of one year at -18 °C [48]. The working solutions of DON, 3-ADON, 15-ADON, DOM-1, DON-3-glucoside and isotope-labelled (^13^C_15_) DON were prepared in methanol and stored at -18 °C. DON-3-glucuronide and DON-15-glucuronide were kindly supplied by Dr. Huybrechts (Sciensano, Tervuren, Belgium). Water was obtained from an Arium^®^ Pro water system from Sartorius (Brussels, Belgium). Disinfectol^®^ (denatured ethanol with 5% ether) was purchased from Chem-Lab (Zedelgem, Belgium). Methanol (LC–MS grade) was supplied by BioSolve (Valkenswaard, the Netherlands), while acetonitrile (Analar Normapur) was purchased from VWR International (Zaventem, Belgium). Formic acid (98–100%) and acetic acid (glacial, 100%) were obtained from Merck (Darmstadt, Germany). Anhydrous magnesium sulphate (>99.5%) was purchased from Alfa Aesar (Haverhill, MA, USA). Sodium chloride (>99.5%) was obtained from VWR Chemicals (Radnor, PA, USA). Anhydrous magnesium sulphate (>99.5%) was purchased from Alfa Aesar (Haverhill, MA, USA) and anhydrous sodium chloride (>99%) from VWR Chemicals (Radnor, PA, USA). Phosphate (PBS) buffer was prepared by dissolving sodium phosphate dibasic and sodium phosphate monobasic in water.

### 4.2. Sample Collection

Based on acquired urinary samples from Vidal et al. [15], the presence of DON and DON metabolites were re-analysed using direct and indirect methodology. Briefly, 20 healthy adults were used as volunteers to perform the study, including 9 men (45%) and 11 women (55%). The average age of the volunteers was 32 years, min 18 and max 61 years old. Sixteen volunteers (four volunteers served as controls) received an oral dose of DON according to the tolerable daily intake (TDI, 1 µg/kg bw/day) and their body weight. Two days prior to and two days after the mycotoxin administration, participants had to follow a scrupulous diet that did not have cereals and cereal-based products. All individual urinary discharges had to be collected during 24 h just after mycotoxin dose intake and stored at −20 °C until analysis. This intervention study was conducted following the guidelines laid down in the declaration of Helsinki and the Ethical Committee of the Ghent University Hospital (B670201630414, approved on 2th February 2017). All individual participants included in this study had to sign an informed consent. Ninety-six urine samples with the highest DON and DON metabolites incidence were selected to perform the current study.

### 4.3. Enzymatic Treatment

For the hydrolysis experiments, 0.25 mL of PBS buffer (pH 7.4) containing ~ 13,000 U of β-glucuronidase (corresponding to approximately 6000 units/mL) was added to 2.1 mL of urine. The samples were incubated overnight at 37 °C. Afterwards, they were centrifuged at 5750× *g* for 10 min, and 2 mL of the supernatant was transferred to another plastic tube. Samples for direct quantification also received 0.25 mL of PBS buffer without enzymatic presence.

### 4.4. Sample Preparation and Targeted LC–MS/MS Analysis

Ten microliters of internal standard was added to 2 mL of pre-treated urine sample and 18 mL of acetonitrile/water/formic acid (52/45/3, *v*/*v*/*v*) and finally 4 g of anhydrous magnesium sulphate and 1 g of sodium. After addition, the sample was forcefully shaken by hand. Later, the samples were collocated on an agitator decanter overhead shaker for 30 min (Agitelec, J. Toulemonde & Cie., Paris, France) and then centrifuged at 4000× *g* for 6 min.

To 18 mL of acetonitrile/water/formic acid (52/45/3, *v*/*v*/*v*), 2 mL of the pre-treated urine sample was added in combination with 10 µL of internal standard, 4 g of anhydrous magnesium sulphate and 1 g of sodium chloride. After addition, the sample was vigorously shaken by hand. Then, the samples were placed on an agitator decanter overhead shaker for 30 min (Agitelec, J. Toulemonde & Cie., Paris, France) and centrifuged at 4000× *g* for 6 min. Seven milliliters of the upper layer (non-polar fraction) was evaporated to dryness using a gentle nitrogen stream at 40 °C. To finish, the residue was redissolved in 250 µL injection solvent (methanol/water, 10/90, *v*/*v*).

The urine samples were analysed using a LC–MS/MS (Waters Acquity UPLC system coupled to a Quattro XEVO TQS mass spectrometer). Data acquisition and processing were performed with MassLynx™ version 4.1 and QuanLynx^®^ version 4.1 software (Waters, Manchester, UK). A column Waters Acquity UPLC^®^ HSS T3 (2.1 × 100 mm, 1.8 µm) was used (Waters, Manchester, UK). Two mobile phases were applied for the analysis. The two mobile phases were water/acetic acid (99.9/0.1, *v*/*v* (A)) and methanol/acetic acid (99.9/0.1, *v*/*v* (B)). The gradient program started at 99% mobile phase A. After an isocratic phase for 0.5 min at initial conditions, mobile phase B increased to 45% in 6 min. Then, a plateau phase for 1.5 min was enhanced with 99% mobile phase B. An equilibration step of 1.5 min was introduced, resulting in a total run time of 9 min. The flow rate was set at 0.4 mL/min. Positive and negative electrospray ionisation modes (ESI+/ESI-) were set off in the mass spectrometer. The capillary voltage was 30 kV, and nitrogen was applied as spray gas. The source and desolvation temperatures were set at 150 °C and 200 °C, respectively. The argon collision gas pressure was 9 × 10^−6^ bar, the cone gas flow 50 L/h and the desolvation gas flow 500 L/h. Two selected product ions with a specific dwell-time were optimized for each analyte, in order to increase the sensitivity and the selectivity of the mass spectrometric conditions (Table 3). DON-3-glucuronide and DON-15-glucuronide had the same MS/MS transitions and exactly the same case for 3-ADON and 15-ADON. The used LC–MS/MS method was validated [15] based on the European Commission Decision 2002/657/EC laying down the rules for the analytical methods to be used in the testing of official samples [49].

### 4.5. Calculations

All obtained results were carried out on molar basis taking the molecular weight of the analytes (DON, 296 g/mol and DON-3-glucuronide and DON-15-glucuronide, 472 g/mol) and the total urinary volume of each sampling point into account. The statistical analysis was performed using the software Microsoft Excel^®^ 2016 and SPSS^®^ 23.0.

## Figures and Tables

**Table 1:** Presence (%), mean recoveries ± standard deviation (nmol) and maximum concentration (nmol) of DON and its metabolites (DON-3-glucuronide and DON-15-glucuronide) in urine samples.

Method	Free DON			DON-3-GLUCURONIDE			DON-15-GLUCURONIDE			Total DON		
	Presence (%) *	Mean ± SD (nmol)	Max. (nmol)	Presence (%)	Mean ± SD (nmol)	Max. (nmol)	Presence (%)	Mean ± SD (nmol)	Max. (nmol)	Presence (%)	Mean ± SD (nmol)	Max. (nmol)
Direct	43	4.18 ± 2.63	18.23	71	7.51 ± 6.98	74.27	94.0	32.76 ± 30.19	147.86	95.2	37.95 ± 37.21	223.57
Indirect	71	23.89 ± 8.61	44.62	33	2.07 ± 1.67	17.74	92.0	8.38 ± 7.60	42.09	95.2	27.69 ± 26.64	150.56

DON = deoxynivalenol; SD = standard deviation; Max.= maximum concentration; Total DON = deoxynivalenol + deoxynivalenol-3-glucuronide + deoxynivalenol-15-glucuronide; * Percentage of samples > LOQ.

**Table 2:** Hydrolysis conditions (pH, temperature, time, type and concentration of the enzyme) and maximum deoxynivalenol concentration found in human urine in the studies where indirect urinary deoxynivalenol analysis was performed.

pH	Time	Enzyme type	Enzyme concentration (units/mL)	Max DON concentration (ng/mL)	Reference
-	18 h	*Helix pomatia* (Type H-2)	6510	67.4	[17]
-	Overnight	*Helix pomatia* (Type H-2)	6510	14.2	[25]
-	Overnight	-	5750	152.6	[26]
-	Overnight	*Helix pomatia* (Type H-2)	6510	353.0	[27]
7.4	18 h	*Escherichia coli* (Type IX)	6000	40.0	[28]
7.4	18 h	*Escherichia coli* (Type IX)	6000	19.5	[23]
5.0	18 h	*Helix pomatia* (Type I)	5000/10,000	26.2	[24]
7.4	18 h	*Escherichia coli* (Type IX)	5000/10,000	26.2	
6.8	18 h	*Patella vulgate* (Type L-II)	5000/10,000	26.2	
-	-	*Escherichia coli* (Type IX)	5750	116.7	[29]
7.4	18 h	-	7000	59.9	[30]
7.4	18 h	*Escherichia coli* (Type IX-A)	4500	7.0	[31]
	Overnight	-	5750	78.2	[32]
7.4	18 h	*Escherichia coli* (Type IX-A)	5750	28.8	[33]
6.8	18 h	*Escherichia coli* (Type IX-A)	5750	10.5	[34]
7.2	18 h	*Escherichia coli* (Type IX-A)	5750	48.2	[35]
-	Overnight	*Helix pomatia* (Type H-2)	6510	72,439.0	[36]
6.8	18 h	*Escherichia coli* (Type IX)	2000	247	[37]
6.8	18 h	*Escherichia coli* (Type IX-A)	23,000	13.8	[38]
6.8	18 h	*Escherichia coli* (Type IX-A)	23,000	140.9	[39]
7.4	Overnight	*Escherichia coli* (Type IX-A)	1750	135.2	[40]
6.8	18 h	*Escherichia coli* (Type IX-A)	23,000	135.0	[41]
5.0	18 h	*Helix pomatia* (Type H-1)	4000	84.1	[42]
5.0	Overnight	*Helix pomatia*	0.044	14.6	[43]
6.8	18 h	*Escherichia coli* (Type IX)	23,000	436.0	[44]
5.0	Overnight	*Helix pomatia*	0.044	1.8	[45]
5.0	Overnight	*Helix pomatia*	0.073	7.2	[46]
-	18 h	*Helix pomatia* (Type H-2)	6510	>20.0	[47]

- Information not present in the manuscript.

**Table 3:** ESI–MS/MS parameters used for the analysis of deoxynivalenol (DON), deoxynivalenol-3-glucoside (DON-3-glucoside), deoxynivalenol-3-glucuronide/deoxynivalenol-15-glucuronide (DON-3-glucuronide/DON-15-glucuronide), 3-acetyldeoxynivalenol/15-acetyldeoxynivalenol (3-ADON/15ADON), de-epoxy-deoxynivalenol (DOM-1) and isotope-labelled (^13^C_15_) deoxynivalenol (^13^C_15_ DON) in urine and the respective limits of detection and quantification.

Mycotoxin	Precursor ion (m/z)	Product ions ^a^ (m/z)	CE ^a,b^ (eV)	CV ^c^ (V)	Retention time (min)	LOD ^d^ (ng/mL)	LOQ ^e^ (ng/mL)
DON	297.0	249.0/231.0	9/9	40	3.99	0.2	0.4
DON-3-glucoside	459.1	168.1/132.0	10/9	15	3.90	0.3	0.6
DON-3-glucuronide/ DON-15-glucuronide	471.0	113.0/193.0	30/24	60	3.65/3.78	0.5	1.0
3-ADON/15-ADON	339.0	231.0/203.1	15/9	15	5.69	0.1	0.2
DOM-1	281.1	215.1/233.1	9/9	40	4.83	0.6	1.2
^13^C_15_ DON	311.9	262.9/130.5	10/10	30	3.99		

^a^ Values are given as quantifier ion/qualifier ion; ^b^ CE: collision energy; ^c^ CV: cone voltage; ^d^ LOD: limit of detection; ^e^ LOQ: limit of quantification.
